# Letter to the editor: “Not all biases are bad: equitable and inequitable biases in machine learning and radiology”

**DOI:** 10.1186/s13244-021-01022-5

**Published:** 2021-06-16

**Authors:** Antoine Iannessi, Hubert Beaumont, Anne Sophie Bertrand

**Affiliations:** 1 Centre Antoine Lacassagne, 33 Avenue de Valombrose, 06100 Nice, France; 2 Median Technologies, 1800 route des crêtes, 06560 Valbonne, France; 3 Centre Hospitalier Princess Grace, 1 Avenue Pasteur, 98000 Monaco, Monaco

**Keywords:** Artificial intelligence, Bias, Inequity

## Abstract

Artificial intelligence algorithms are booming in medicine, and the question of biases induced or perpetuated by these tools is a very important topic. There is a greater risk of these biases in radiology, which is now the primary diagnostic tool in modern treatment. Some authors have recently proposed an analysis framework for social inequalities and the biases at risk of being introduced into future algorithms. In our paper, we comment on the different strategies for resolving these biases. We warn that there is an even greater risk in mixing the notion of equity, the definition of which is socio-political, into the design stages of these algorithms. We believe that rather than being beneficial, this could in fact harm the main purpose of these artificial intelligence tools, which is the care of the patient.

## Keypoints

- ‘Health equity’ terminology is socio-politically based, committed to eliminating disparities in health.
- The patient’s medical interest should prevail over social equity in debiasing strategy.
- Transparency in artificial intelligence can debias by providing contextualized information to radiologists.

## Background

Dear Editor in Chief,

We read with interest the article entitled ‘Not all biases are bad: equitable and inequitable biases in machine learning and radiology’ by Pot et al. [1] recently published in* Insights into Imaging*.

The authors propose a framework to analyze how social inequities in health transition into artificial intelligence (AI) algorithms in radiology. To illustrate their topic, they use race, gender and wealth inequities as examples, drawing a parallel between the root of such unfair inequalities and potential biases existing in machine learning (ML) radiology. They state that distributive and relational inequities are at risk of being translated into dataset bias quantitatively and qualitatively, respectively. Moreover, they are concerned about specific ‘socially related’ cognitive biases transiting into ML algorithms.

## Main text

To better understand what is as stake, ‘health equity’ must be understood as a political terminology and the principle underlying a commitment to eliminate disparities in health and its determinants, including social determinants [2].

Indeed, there is unequivocal strong evidence to link economic/social disadvantage with lack of healthcare opportunities, illness and disability. These inequalities are also unfair, because they could be reduced by the right mix of government policies, according to the World Health Organization [3].

Additionally, the authors are concerned with cultural bias (i.e., the interpretation of situations, actions or data based on the standards of one's own culture). This bias is discriminative because it is associated with partiality to a sub-group value. Cognitive biases regroup under different names and have been previously described in radiology as attribution biases, as mentioned by the authors.

We agree with the authors that AI systems for radiology are not free of bias, which can be deleterious or useful, and that engineers, radiologists and politicians should be aware of bias when developing/using AI algorithms.

However, our opinions differ on how to consider and manage bias. We are aware of the emergence of equally discriminatory strategies in the fight against cultural bias, which we believe are not the best approach to tackling the issue.

Indeed, debias strategies discussed by the authors include introducing another bias to compensate for the one identified as being at risk of contaminating the AI algorithm in radiology. They suggest that a ‘better’ ratio of ethnicity, level of wealth or patient gender must be enforced in the dataset considered, to balance a ‘socially inequitable’ distribution. To handle qualitative cognitive biases, they suggest that one could positively discriminate algorithm developers or involved radiologists in order to promote a diversity of opinion.

This approach can be criticized for three major reasons:

- First, voluntarily injecting a correction inside the ML algorithm in radiology is driven by political motives.
- Second, if these corrections are not transparent to the end user, an additional bias would be introduced, which is not consistent with the initial objective. Indeed, unlike the systematic error controlled by the developer when training the algorithm, these biases are not perceived by the radiologist and are therefore very difficult to avoid without awareness.
- Third, enforcing homogeneity across identified subpopulations in the training data can lead to risky and uncontrolled situations. Unless evidence can be collected to the contrary, this runs the risk of jeopardizing the performance of the ML algorithm for other/unidentified subpopulations, when applied to the general population. This seems to be at odds with the primary objective of offering the most medically efficient algorithm possible to a patient as a non-political individual. Indeed, in a perspective to market an algorithm over a large territory, we believe that the population of validation should be representative of the population of utilization, thus limiting the ‘equitable generalization process’.

Ultimately, collecting ‘equitable’ data for an ML algorithm is a political concern and, in our opinion, should not be considered without evidence of increased patient benefit. It is also essential that the end user radiologist is fully aware of any such corrections, if applied.

**Fig. 1:**
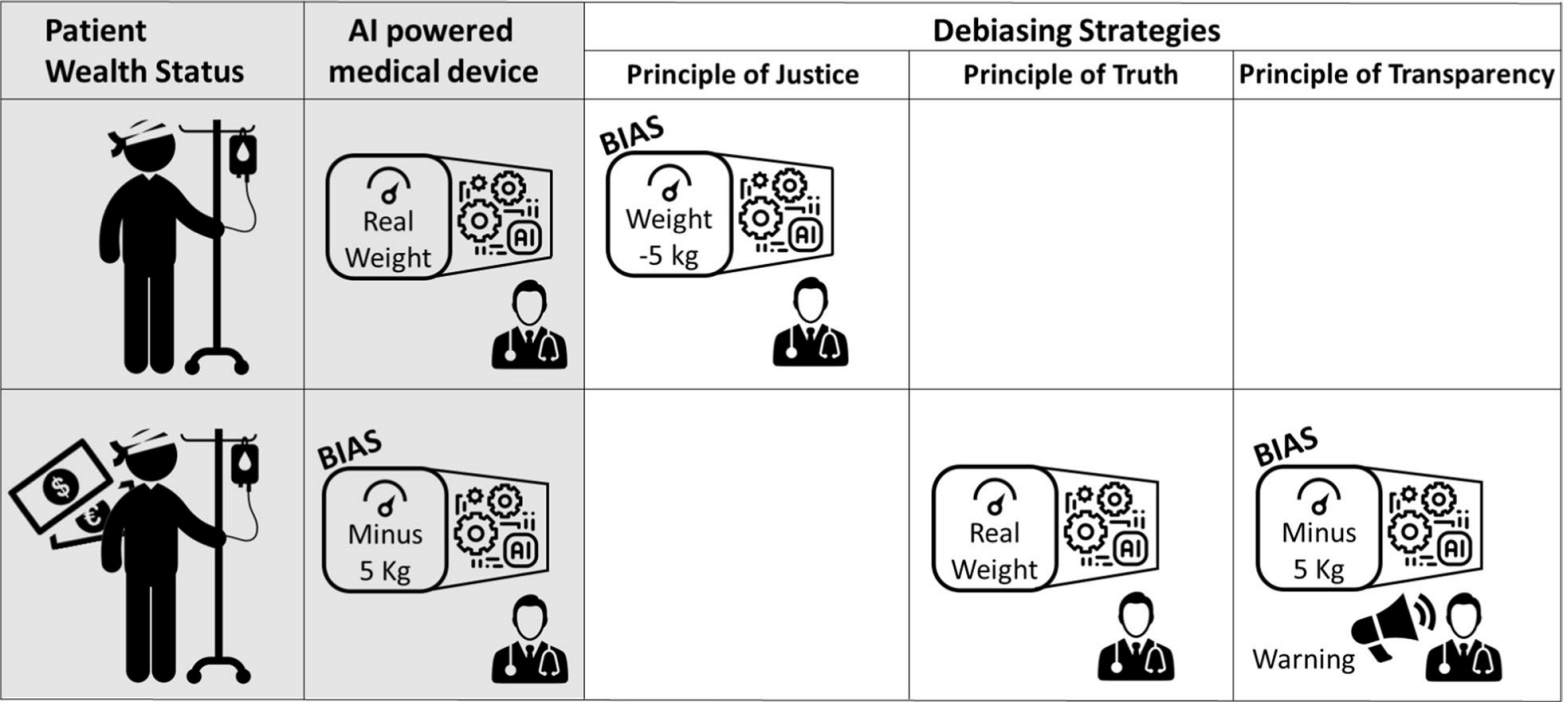
An artificial intelligence powered diagnostic tool in radiology presented as a weighing scale for understanding. The tool reduces the weight of a rich person by 5 kg but uses the actual weight of a poor person. It is unfairly biased in favor of rich people leading to these patients being diagnosed as overweight less frequently. To resolve the bias, if one prioritizes the principle of equity, and reduces the weight of a poor person by 5 kg as well, the outcome of the scale is wrong for both groups of patients. If one focuses on obtaining a correct weight for all the patients, one can cancel the error on the biased scale or inform the user that the result may be biased. In conclusion, in terms of debiasing strategy, the patient’s medical interest prevails over the principle of social equity. *AI* Artificial intelligence

As an analogy, we can take the example of a weighing scale which systematically reduces weight by 5 kg for a rich person but not for a poor person (Fig. 1). If your decision is based on weight, you would reduce the weight by 5 kg for a poor person as well if following a principle of justice, or measure the actual weight of a rich person, if following a principle of truth. If you cannot correct the scale, the principle of transparency applies, and you should advertise the risk of an erroneous result for rich people [4].

Medicine is a branch of human science and is based on an ideal of neutrality. Regarding the social-related cognitive bias applying to radiologists facing inequities, we would like to refer to the original Hippocratic oath, i.e., ‘Into whatever homes I go, I will enter them for the benefit of the sick, avoiding any voluntary act of impropriety or corruption’ [5]. Swearing this oath does not eliminate bias in social individuals (radiologists included), but we believe that physicians fundamentally respect this oath by treating all patients equally.

## Conclusion

In conclusion, we believe the strategies for debias suggested by the authors will not help solve the problem, and that radiology should be kept away from political interference. Social and cultural biases are deeply political, and we agree with the authors that there is a risk of such bias creeping into newly built algorithms in radiology. Such bias is supported by the concept of inequities, leading us to think that a good solution for debias would be to restore the equity. As we have explained, the concept of ‘debiasing’ does not mean ‘to compensate for’ but ‘to remove’ the bias. Therefore, we do not believe that there should be an aim to compensate for social or cultural bias in the conception of AI in radiology. The only possible relevant exception would be if the outcome of the algorithm results in increased medical benefit to the patient. Even then, the user should be fully aware that the algorithm will propose a diagnostic based on a ‘corrected’ population so that they can make an informed decision.

## Data Availability

No specific data were used.

## Abbreviations

AI: Artificial intelligence
ML: Machine learning
